# The expression of the insulin receptor in gastric cancer correlates with the HER2 status and may have putative therapeutic implications

**DOI:** 10.1007/s10120-019-00964-6

**Published:** 2019-04-15

**Authors:** Steffen M. Heckl, Viva Wiesener, Hans-Michael Behrens, Dita Ulase, Sandra Krüger, Christoph Röcken

**Affiliations:** 1 Department of Pathology, Christian-Albrechts-University, Arnold-Heller-Str. 3, Haus 14, 24105 Kiel, Germany; 2 Department of Internal Medicine I, University Hospital Schleswig–Holstein, Kiel, Germany; 3 Department of Pathology, Riga Stradins University, Riga, Latvia

**Keywords:** Gastric cancer, Insulin receptor, HER-2 Proto-Oncogene Protein, Prognosis, Cancer treatment

## Abstract

**Background:**

Metabolic reprogramming in gastric cancer (GC) involves not only an alteration of glucose metabolism, but also of insulin receptor (IR) expression. We investigated if (1) GCs express the IR in cancer cells (CC-IR) and vasculature (VIR), (2) IR expression is clinically relevant and may be a novel target of GC treatment.

**Methods:**

467 primary GCs were studied by immunohistochemistry using an IR-specific antibody. CD31-immunostaining ensured the presence of representative intratumoral microvasculature. VIR, and membranous and cytoplasmic CC-IR (mCC-IR, cCC-IR) were evaluated using a modified HistoScore (HScore) and subsequently dichotomized into low or high IR expressions. The IR status was correlated with clinico-pathological patient characteristics, including survival and HER2 status.

**Results:**

VIR, mCC-IR, and cCC-IR (HScore > 0) were found in 97.0%, 87.6%, and 95.7% of all GCs. After dichotomization of the HScores, 50.7, 48.8, and 50.3% were classified as VIR-high, mCC-IR-high, and cCC-IR-high, respectively. IR was associated with the Laurén phenotype, tumor localization, local tumor growth, vascular invasion, perineural invasion, tumor budding, mucin phenotype, UICC stage, worse survival, and the HER2 status. On multivariate analysis, VIR status was an independent prognosticator of overall (*p* = 0.010) and tumor-specific (*p* = 0.006) patient survival.

**Conclusions:**

VIR and CC-IR expressions are frequent in GC, biologically significant and even correlate with the HER2 status, opening avenues for novel putative therapeutic interventions in GC.

## Background

The hallmarks of cancer enclose six biological capabilities, i.e., sustained proliferative signaling, evasion of growth suppressors, resistance to cell death, replicative immortality, angiogenesis, and activation of invasion and metastasis. In 2011, Hanahan and Weinberg amended their hallmarks and added, e.g., reprogramming of energy metabolism [1]. Metabolic reprogramming could also be regarded as an underlying concept in gastric cancer (GC) [2]: one cornerstone of metabolic reprogramming comprises an increased glucose uptake and metabolism in cancer cells [2], which is utilized in PET imaging [3]. Barely studied for GC until now, metabolic reprogramming also involves an altered expression of the insulin receptor (IR).

The IR is a transmembrane receptor of the receptor tyrosine class, which is located at the cell surface and in cytoplasmic vesicles. It co-governs energy regulation and stimulates proliferation [4]. The two isoforms of the IR convey different functionalities: metabolic effects are exerted via isoform B (IR-B) [4]. IR-B preferentially binds insulin. The isoform A (IR-A) is overexpressed in cancer, and provides mitogenic and proliferative effects [4]. The IR-A binds insulin and IGF-II. An increased expression of the IR in cancer cells (CC-IR) has been described in renal, colorectal, breast, lung, thyroid, and ovarian cancers [4]: interestingly, in renal cancer, CC-IR was found to be associated with a prolonged disease-free and overall survival [5]. In breast cancer, the correlation between CC-IR and patient survival led to opposing results [6, 7]. In non-small cell lung cancer, the expression of IR in tumor cells was associated with poor patient outcome [8].

In this study, we tested the following hypotheses: (1) GCs express the IR in tumor cells (CC-IR) and tumor vasculature (VIR). (2) The expression of the IR is biologically relevant and may provide a novel target of GC treatment.

## Methods

### Study population and histology

From the archive of the Institute of Pathology, University Hospital Schleswig–Holstein, Kiel, we sought all patients who had undergone either total or partial gastrectomy for adenocarcinoma of the stomach or esophago-gastric junction between 1997 and 2009. All tissue samples originated from routine therapeutic surgeries, for all of which the patients had given written informed consent. Ethical approval was obtained from the local ethical review board (D 453/10 and D 468/17) of the University Hospital Schleswig–Holstein, Kiel, Germany, which permitted us to use the samples from those patients who had also given written informed consent for a prospective scientific use of their patient material. All patient data were pseudonymized after study inclusion. The following patient characteristics were retrieved: type of surgery, age at diagnosis, gender, tumor size, tumor localization, tumor type, depth of invasion, number of lymph nodes resected, and number of lymph nodes with metastases. Patients were included if an adenocarcinoma of the stomach or esophago-gastric junction was histologically confirmed. Exclusion criteria were defined as (1) histology identified a tumor type other than adenocarcinoma, and (2) patients had undergone a perioperative or neoadjuvant chemo- or radiotherapy. Each resected specimen had undergone gross sectioning and histological examination by trained and board-certified surgical pathologists. Date of patient death and cause of death were obtained from the Epidemiological Cancer Registry of the state of Schleswig-Holstein, Germany, thereby distinguishing between tumor-related deaths and deaths from other causes. Follow-up data of those patients who were still alive were retrieved from hospital records and general practitioners.

### Histology

Tissue specimens were fixed in formalin and embedded in paraffin. Deparaffinized sections were stained with hematoxylin and eosin. Histological re-examination of primary tissue sections was carried out for all cases to assure if inclusion criteria were met. Tumors were classified according to the Laurén classification [9] and re-examined by two surgical pathologists. pTNM stage of all study patients was determined according to the 8th edition of the UICC guidelines [10].

### Tumor budding

Tumor budding was assessed on H&E-stained specimens, following the recommendations of the International Tumor Budding Consensus [11], which defined budding as “a single tumor cell or a cell cluster of up to 4 tumor cells” [11]. For each patient sample, the H&E slide with the most pronounced tumor budding at the invasion front was identified. Within the chosen slide, ten separate fields were screened using a 10× objective, to identify the tumor budding “hotspot”. Employing the “hotspot” approach, tumor buds were counted within one field of a “hotspot” area using a 20× objective lens with a 23 mm eyepiece field number diameter. The tumor bud count was normalized by the factor 1.323 to correspond the standardized field area of 0.785 mm^2^. Tumor budding was evaluated as being absent (Bd0; 0 buds), 1–4 buds (Bd1), 5–9 buds (Bd2), and ≥ 10 buds (Bd3) per standardized field area. Similar to Kemi et al. [12], we then categorized tumor budding into three groups, i.e., no (Bd0; 0 buds), low (Bd1 and Bd2; < 10 buds) and high tumor budding (Bd3; ≥ 10 buds). We added the Bd0 (no buds) group, which is not part of the International Tumor Budding Consensus, as we consider it as an own group.

### Immunohistochemistry

Immunohistochemistry was carried out with monoclonal antibodies directed against CD31 (dilution 1:100; mouse monoclonal antibody; JC70; Cell Marque, California, USA), mucin 2 (dilution 1:100; mouse monoclonal antibody; clone Ccp58; Novocastra; Leica Microsystems GmbH, Wetzlar, Germany), mucin 5 (dilution 1:100; mouse monoclonal antibody; clone 45M1; Thermo Scientific, Schwerte, Germany), and CD10 (dilution 1:10; mouse monoclonal antibody; clone 56C6; Novocastra; Leica Microsystems GmbH, Wetzlar, Germany).

Immunostaining was performed with the autostainer Bond™ Max System (Leica Microsystems GmbH, Wetzlar, Germany): Antigen retrieval was done with ER1 (citrate buffer Bond pH 6.0; CD10), or ER2 (EDTA-buffer Bond pH 9.0; CD31, mucin 2) according to the manufacturer`s instructions. The mucin 5 immunostaining did not necessitate any antigen retrieval. The immunoreaction was visualized with the Bond™ Polymer Refine Detection Kit (DS 9800; brown labeling; Novocastra; Leica Microsystems GmbH, Wetzlar, Germany).

IR immunostaining was carried out manually using a rabbit monoclonal anti-insulin receptor β-antibody (dilution 1:50; clone 4B8; Cell Signaling Technologies, Danvers, USA), which detects both insulin receptor isoforms: deparaffinized tissue sections were boiled in EDTA buffer (pH 9.0; 1 min; 125 °C) and then washed with Tris-buffered saline (TBS), blocked with hydrogen peroxide block (Thermo Fisher Scientific) for 15 min, washed with TBS, and then treated with Ultra V Block (Thermo Fisher Scientific) for 5 min. Immunoreactions were visualized with the ImmPRESS reagent peroxidase universal anti-mouse/rabbit Ig—MP-7500 and the ImmPact NovaRed peroxidase substrate SK-4805 Kit (Vector Laboratories, Burlingame, CA, USA, respectively). Immunohistochemistry was followed by counterstaining with hematoxylin. Negative controls were generated by omitting the primary antibody. Healthy endometrial tissue specimens (proliferative phase) served as positive controls.

### Evaluation of CD31 and IR immunostaining

The CD31-immunostaining served to prove the presence of intratumoral vessels, especially the presence of capillaries, within the respective tumor samples. Cancer vessels were defined as capillaries, arterioles, and venules being surrounded by cancer cells.

Immunostaining of the IR was evaluated according to the modified HistoScore (HScore). The first parameter was based on the intensity of the stained cells. A score of 0 (no evidence of staining), 1 + (weak), and 2 + (strong staining reaction) was applied. The second parameter assessed the percentage of cells showing no (0), weak (1+), or strong (2+) immunostaining. In each case, the percentages add up to 100%: a case lacking any expression of IR would be categorized as 100% negative and a case which showed strong immunostaining of half of the tumor cells and no immunostaining of the reminder would be categorized as 50% negative (0) and 50% strongly positive (2+). Using this approach, intratumoral heterogeneity was readily evident.

Finally, an HScore was calculated according to the formula: HScore = [0 × percentage of immunonegative tumor cells] + [1 × percentage of weakly stained tumor cells] + [2 × percentage of strongly stained tumor cells]. The maximum possible HScore was 200, if all cells of a given tumor sample showed a strong staining: [0 × 0%] + [1 × 0%] + [2 × 100%] = 200. The multipliers within the formula yield an improved stratification of the HScores: tumor samples with a predominantly high staining intensity and such samples with a predominantly low staining intensity are more distinctively separated by the HScores.

**Fig. 1:**
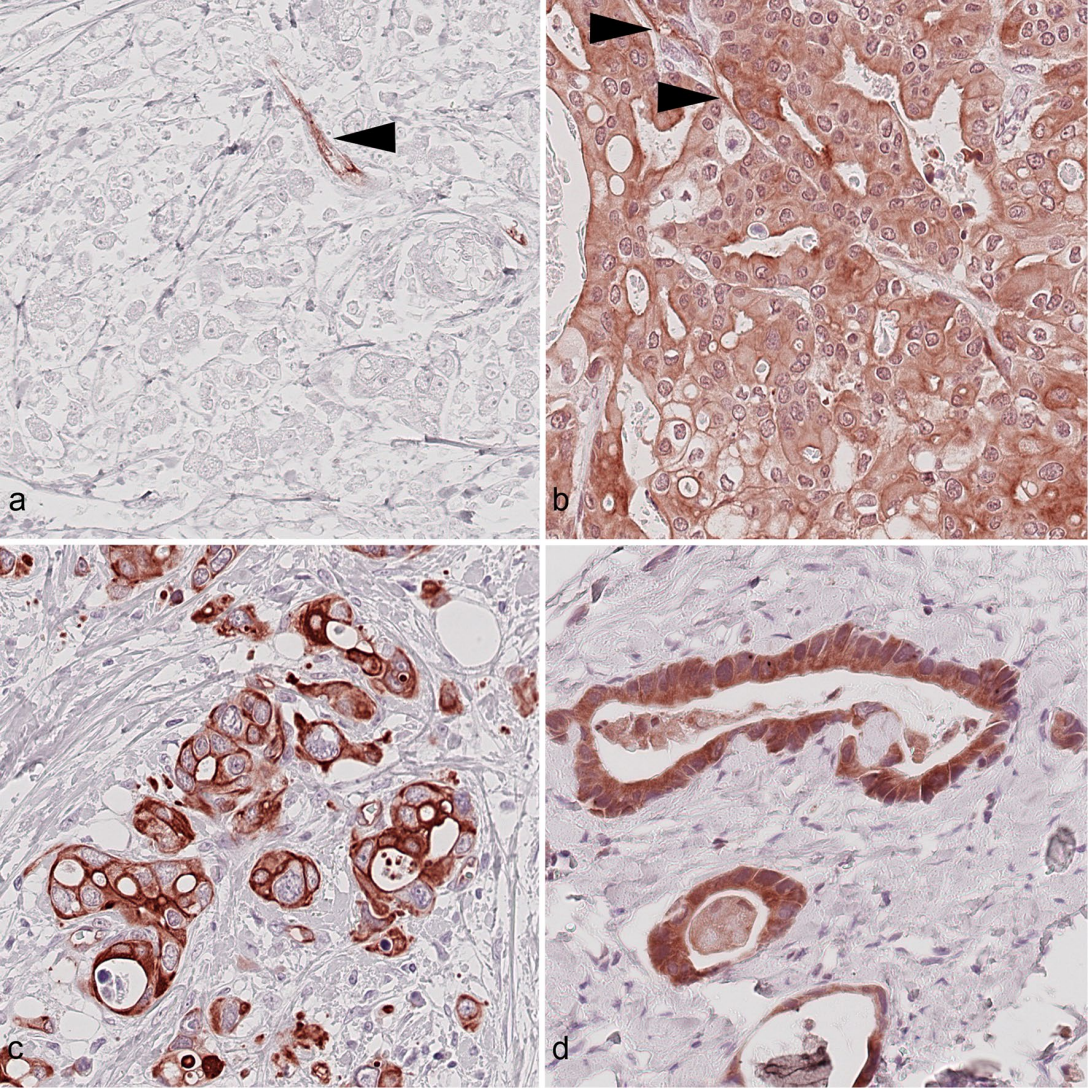
Expression of the insulin receptor in gastric cancer. Gastric cancer samples showing, **a** high vascular (VIR-high, HScore ≥ 115; arrow head) insulin receptor staining without immunostaining of cancer cells, **b** high cytoplasmic (cCC-IR-high, HScore ≥ 90), high vascular (VIR-high, HScore ≥ 115; arrow heads), but low membranous insulin receptor staining (mCC-IR-low, HScore ≤ 40). Examples of **c** high membranous insulin receptor expression (mCC-IR-high, HScore > 40), but low cytoplasmic insulin receptor expression in tumor cells (cCC-IR-low, HScore < 90) and of **d** high cytoplasmic insulin receptor expression (cCC-IR-high, HScore ≥ 90), but no membranous insulin receptor expression in tumor cells (mCC-IR-low, HScore ≤ 40). Anti-insulin receptor immunostaining, hematoxylin counterstaining. Original magnification A-F: 400×

The entire series was screened and three representative cases were selected for the adjustment of IR 0, IR 1+, and IR 2+ staining intensity (Fig. 1). These cases were subsequently used as reference standards for the in-depth evaluation of the entire cohort.

IR immunostaining was evaluated separately for cytoplasmic (cCC-IR) and membranous expression (mCC-IR) in cancer cells, and for endothelial cells of cancer vessels (VIR). Finally, the median HScore served as a cut-off to differentiate between high and low IR expression.

### Assessment of mucin phenotype

The classification of the mucin phenotype was done according to Namikawa et al. [13]: We distinguished the gastric (MUC5+ , CD10−, MUC2−), intestinal (MUC5−, CD10+ , MUC2± or MUC5−, CD10−, MUC2+), combined (MUC5+, CD10+, MUC2± or MUC5+ , CD10−, MUC2+), and unclassified type (MUC5−, CD10−, MUC2−).

### Assessment of the HER2 status

The HER2 status was assessed as previously described [14]. In brief, a monoclonal anti-Her2/neu antibody (clone 4B5 with a Ventana BenchMark XT automated slide staining system, both Roche Diagnostics GmbH, Mannheim, Germany) was used for immunohistochemistry: the immunostaining intensity was scored ranging from negative (0) to strong (3+). For all GC cases with an immunostaining of 2+ , a silver-enhanced in situ hybridization was performed (HER2-SISH double-labeling in situ hybridization system; Roche Diagnostics GmbH, Mannheim, Germany). If a strong immunostaining (3+) was present in ≥ 10% of the tumor area or a moderate immunostaining (2+) together with an HER2 gene amplification (ratio ≥ 2.0), the sample was classified as HER2 positive.

### Statistical analyses

SPSS version 24.0 (IBM Corp., Armonk, NY, USA) was used for statistical analyses. The correlation between non-ordinal clinico-pathological patient characteristics and the VIR status or the CC-IR status was tested with Fisher’s exact test. T category, N category, UICC stage, tumor grading, and tumor budding as variables of ordinal scale were tested with Kendall’s tau test. Median survival with 95% confidence intervals was determined by the Kaplan–Meier method. Differences between median survivals were tested with the log-rank test. A multivariate survival analysis (Cox regression) was performed. A *p* value of ≤ 0.05 was considered to be significant. All p values are given uncorrected. To compensate false discovery rate within the correlations, we applied the Siemes (Benjamini-Hochberg) procedure. P values having lost significance are marked.

## Results

### Study population

Table 1 summarizes the clinico-pathological patient characteristics of the GC cohort. 467 patients fulfilled all study criteria.

**Table 1:** Correlation of clinico-pathological patient characteristics with insulin receptor expression in tumor cells and tumor vessels

			Total	IR vascular expression		IR cancer cell expression: cytoplasm		IR cancer cell expression: membranous	
				Low: HScore, < 115	High: HScore, ≥ 115	Low: HScore, < 90	High: HScore, ≥ 90	Low: HScore, ≤ 40	high: HScore, > 40
			*n* (%)	*n* (%)	*n* (%)	*n* (%)	*n* (%)	*n* (%)	*n* (%)
Gender	*n*	*p* value^a^	467	467	0.027*	467	0.848	467	0.335
Male			298 (63.8)	135 (45.3)	163 (54.7)	147 (49.3)	151 (50.7)	158 (53.0)	140 (47.0)
Female			169 (36.2)	95 (56.2)	74 (43.8)	85 (50.3)	84 (49.7)	81 (47.9)	88 (52.1)
Age group	*n*	*p* value^a^	461	461	0.352	461	0.005	461	0.136
< 68 years			231 (50.1)	119 (51.5)	112 (48.5)	129 (55.8)	102 (44.2)	126 (54.5)	105 (45.5)
≥ 68 years			230 (49.9)	108 (47.0)	122 (53.0)	98 (42.6)	132 (57.4)	109 (47.4)	121 (52.6)
Lauren	*n*	*p* value^a^	467	467	< 0.001	467	< 0.001	467	0.043*
Intestinal			243 (52.0)	101 (41.6)	142 (58.4)	84 (34.6)	159 (65.4)	110 (45.3)	133 (54.7)
Diffuse			142 (30.4)	100 (70.4)	42 (29.6)	115 (81.0)	27 (19.0)	79 (55.6)	63 (44.4)
Mixed			32 (6.9)	16 (50.0)	16 (50.0)	16 (50.0)	16 (50.0)	18 (56.3)	14 (43.8)
Unclassified			50 (10.7)	13 (26.0)	37 (74.0)	17 (34.0)	33 (66.0)	32 (64.0)	18 (36.0)
Mucin phenotype	*n*	*p* value^a^	406	406	0.022*	406	0.065	406	0.740
Intestinal type			117 (28.8)	45 (38.5)	72 (61.5)	47 (40.2)	70 (59.8)	65 (55.6)	52 (44.4)
Gastric type			59 (14.5)	36 (61.0)	23 (39.0)	34 (57.6)	25 (42.4)	30 (50.8)	29 (49.2)
Combined type			158 (38.9)	72 (45.6)	86 (54.4)	77 (48.7)	81 (51.3)	77 (48.7)	81 (51.3)
Unclassified			72 (17.8)	39 (54.2)	33 (45.8)	41 (56.9)	31 (43.1)	37 (51.4)	35 (48.6)
Localization	*n*	*p* value^a^	454	454	0.001	454	0.015*	454	0.839
Proximal stomach			140 (30.8)	52 (37.1)	88 (62.9)	57 (40.7)	83 (59.3)	72 (51.4)	68 (48.6)
Distal stomach			314 (69.2)	171 (54.5)	143 (45.5)	168 (53.5)	146 (46.5)	157 (50.0)	157 (50.0)
T category	*n*	*p* value^b^	466	466	0.002	466	0.146	466	0.074
T1a/T1b			55 (11.8)	42 (76.4)	13 (23.6)	24 (43.6)	31 (56.4)	30 (54.5)	25 (45.5)
T2			54 (11.6)	28 (51.9)	26 (48.1)	19 (35.2)	35 (64.8)	29 (53.7)	25 (46.3)
T3			185 (39.7)	83 (44.9)	102 (55.1)	100 (54.1)	85 (45.9)	102 (55.1)	83 (44.9)
T4a/T4b			172 (36.9)	76 (44.2)	96 (55.8)	88 (51.2)	84 (48.8)	77 (44.8)	95 (55.2)
N category	*n*	*p* value^b^	464	464	0.019*	464	0.208	464	0.473
N0			131 (28.2)	79 (60.3)	52 (39.7)	61 (46.6)	70 (53.4)	69 (52.7)	62 (47.3)
N1			62 (13.4)	27 (43.5)	35 (56.5)	27 (43.5)	35 (56.5)	31 (50.0)	31 (50.0)
N2			85 (18.3)	40 (47.1)	45 (52.9)	44 (51.8)	41 (48.2)	46 (54.1)	39 (45.9)
N3a/N3b			186 (40.1)	84 (45.2)	102 (54.8)	98 (52.7)	88 (47.3)	90 (48.4)	96 (51.6)
M category	*n*	*p* value^a^	467	467	0.563	467	0.298	467	0.419
M0			374 (80.1)	187 (50.0)	187 (50.0)	181 (48.4)	193 (51.6)	195 (52.1)	179 (47.9)
M1			93 (19.9)	43 (46.2)	50 (53.8)	51 (54.8)	42 (45.2)	44 (47.3)	49 (52.7)
UICC stage (8th edition)	*n*	*p* value^b^	464	464	0.010	464	0.134	464	0.338
IA/IB			75 (16.2)	53 (70.7)	22 (29.3)	31 (41.3)	44 (58.7)	41 (54.7)	34 (45.3)
IIA/IIB			100 (21.6)	45 (45.0)	55 (55.0)	50 (50.0)	50 (50.0)	52 (52.0)	48 (48.0)
IIIA/IIIB/IIIC			196 (42.2)	88 (44.9)	108 (55.1)	97 (49.5)	99 (50.5)	99 (50.5)	97 (49.5)
IV			93 (20.0)	43 (46.2)	50 (53.8)	51 (54.8)	42 (45.2)	44 (47.3)	49 (52.7)
LN ratio group	*n*	*p* value^a^	464	464	0.193	464	0.033*	464	0.781
< 0.189			223 (48.1)	118 (52.9)	105 (47.1)	99 (44.4)	124 (55.6)	115 (51.6)	108 (48.8)
≥ 0.189			241 (51.9)	112 (46.5)	129 (53.5)	131 (54.4)	110 (45.6)	121 (50.2)	120 (49.8)
L category	*n*	*p* value^a^	432	432	0.067	432	0.442	432	0.178
L0			209 (48.4)	111 (53.1)	98 (46.9)	97 (46.4)	112 (53.6)	112 (53.6)	97 (46.6)
L1			223 (51.6)	98 43.9	125 (56.1)	112 (50.2)	111 (49.8)	104 (46.6)	119 (53.4)
V category	*n*	*p* value^a^	431	431	0.005	431	0.359	431	0.361
V0			383 (88.9)	196 (51.2)	187 (48.8)	189 (49.3)	194 (50.7)	196 (51.2)	187 (48.8)
V1			48 (11.1)	14 (29.2)	34 (70.8)	20 (41.7)	28 (58.3)	21 (43.8)	27 (56.3)
Pn category	*n*	*p* value^a^	432	432	0.771	432	0.005	432	0.175
Pn0			189 (43.8)	94 (49.7)	95 (50.3)	79 (41.8)	110 (58.2)	101 (53.4)	88 (46.6)
Pn1			243 (56.2)	117 (48.1)	126 (51.9)	135 (55.6)	108 (44.4)	113 (46.5)	130 (53.5)
Tumor budding (Bd)	*n*	*p* value^b^	426	426	0.005	426	< 0.001	426	0.227
Bd0			106 (24.9)	52 (49.1)	54 (50.9)	39 (36.8)	67 (63.2)	58 (54.7)	48 (45.3)
Bd1/2			97 (22.8)	28 (28.9)	69 (71.1)	33 (34.0)	64 (66.0)	31 (32.0)	66 (68.0)
Bd3			223 (52.3)	130 (58.3)	93 (41.7)	139 (62.3)	84 (37.7)	123 (55.2)	100 (44.8)
Grading	*n*	*p* value^a^	463	463	0.272	463	< 0.001	463	0.187
Low grade (G1/G2)			108 (23.3)	48 (44.4)	60 (55.6)	37 (34.3)	71 (65.7)	49 (45.4)	59 (54.6)
High grade (G3/G4)			355 (76.7)	181 (51.0)	174 (49.0)	193 (54.4)	162 (45.6)	188 (53.0)	167 (47.0)
R status	*n*	*p* value^a^	448	448	0.889	448	0.166	448	0.209
R0			389 (86.8)	191 (49.1)	198 (50.9)	186 (47.8)	203 (52.2)	201 (51.7)	188 (48.3)
R1/R2			59 (13.2)	28 (47.5)	31 (52.5)	34 (57.6)	25 (42.4)	25 (42.4)	34 (57.6)
HER2 status	*n*	*p* value^a^	423	423	0.478	423	0.002	423	0.073
HER2 negative			389 (92.0)	187 (48.1)	202 (51.9)	200 (51.4)	189 (48.6)	203 52.2	186 (47.8)
HER2 positive			34 (8.0)	14 (41.2)	20 (58.8)	8 (23.5)	26 76.5	12 35.3	22 (64.7)
Overall survival [months]		*p* value^c^			0.044*		0.214		0.026*
Total/events/censored			448/352/96	216/160/56	232/192/40	222/177/45	226/175/51	228/168/60	220/184/36
Median survival			14.1	16.7	12.1	15.5	13.5	17.3	12.5
95% C.I.			12. 1–16.1	11.7–21.6	9.5–14.8	12.7–18.3	10.8–16.3	12.6–21.9	10.1–14.9
Tumor-specific survival [months]		*p* value^c^			0.045*		0.135		0.010
Total/events/censored			421/290/131	199/128/71	222/162/60	208/149/59	213/141/72	218/136/82	203/154/49
Median survival			15.5	19.9	13.4	16.0	14.9	20.3	12.8
95% C.I.			12.7–18.3	14.3–25.6	9.8–17.1	11.8–20.2	10.9–18.9	14.8–25.8	10.1–15.8

^a^Fisher’s exact test

^b^Kendall’s tau test

^c^log-rank test

*p values having lost significance according to the Siemes (Benjamini–Hochberg) procedure for multiple testing

### Immunohistochemistry

IR expression was studied using whole tissue sections.

A weak cytoplasmic immunostaining of tumor cells (cCC-IR 1+) was found in 431 (92.3%) cases and a strong cytoplasmic (cCC-IR 2+) in 254 (54.4%) cases. Immunonegative tumor cells (cCC-IR 0) were found in 364 (78.0%) cases. The percentage area of the three immunostaining cytoplasmic categories, i.e., cCC-IR 0, 1+, and 2+ ranged from 0 to 100%, and the combination of the staining categories in each individual case varied: 20 (4.3%) GCs were completely devoid of any cCC-IR expression. Six cases showed 100% cCC-IR 2+. 441 (94.4%) cases showed various combinations of two or three staining intensities in diverse combinations. The median HScore for cCC-IR was 90 (range 0–200) and the cohort was dichotomized into cCC-IR-low (HScore < 90) and cCC-IR-high (≥ 90) (Fig. 1). 232 (49.7%) GCs were cCC-IR-low and 235 (50.3%) GCs cCC-IR-high.

With regard to membranous immunostaining (mCC-IR), a weak staining (mCC-IR 1+) was found in 343 (73.4%) and a strong (mCC-IR 2+) in 322 (69.0%) cases. Immunonegative tumor cells (mCC-IR 0) were found in 457 (97.9%) cases. The percentage area of the three immunostaining membranous categories, i.e., mCC-IR 0, 1+, and 2+ ranged from 0 to 100%, and the combination of the staining categories in each individual case varied: 58 (12.4%) GCs were completely devoid of any mCC-IR expression. Two cases showed 100% mCC-IR 2+. 407 cases showed various combinations of two or three staining intensities. The median HScore for mCC-IR was 40 (range 0–200), and the cohort was dichotomized into mCC-IR-low (HScore < 40) and mCC-IR-high (≥ 40) (Fig. 1). 239 (51.2%) GCs were mCC-IR-low and 228 (48.8%) mCC-IR-high.

All tumor samples contained tumor capillaries as verified by CD31 immunostaining. We found the insulin receptor to be particularly expressed in the capillaries and only to a lesser extent in arterioles or venules. Vascular insulin receptor expression was always restricted to the cancer site and was never found within adjacent non-neoplastic gastric tissue. A weak immunostaining (VIR 1+) was found in 428 (91.6%) and a strong immunolabeling (VIR 2+) in 361 (77.3%) cases. 14 (3.0%) GCs were VIR 0 despite the presence of CD31-positive endothelial cells. The median HScore was 115 (range 0–200). The cohort was dichotomized into VIR-low (HScore < 115) and VIR-high (≥ 115) (Fig. 1). 230 (49.3%) GCs were VIR-low and 237 (50.7%) VIR-high.

Collectively, these data show that the expression (= combination of intensity of immunostaining and amount of immunopositive tumor areas) of cCC-IR, mCC-IR, and VIR is heterogeneous in GC including “gray-scale” as well as “black-and-white”-immunostaining patterns.

### Correlation of insulin receptor expression in cancer cells and vessels

Expression of VIR, cCC-IR, and mCC-IR were positively associated: VIR-high was synchronously observed with cCC-IR-high in 62.0% (*p* < 0.001) and with mCC-IR-high in 55.3% (*p* = 0.005). cCC-IR-high and mCC-IR-high were simultaneously seen in 57.0% (p < 0.001).

### Correlation of insulin receptor expression in cancer cells (CC-IR) with clinico-pathological patient characteristics

To explore the putative biological significance of IR expression in GC cells, we correlated cCC-IR and mCC-IR with various clinico-pathological patient characteristics (Table 1). GC patients with cCC-IR-high were significantly older and had more commonly an intestinal or differentiated phenotype of lower tumor grade. Interestingly, cCC-IR also correlated inversely with tumor budding and perineural invasion (Table 1).

No other significant associations between either cCC-IR or mCC-IR and clinico-pathological patient characteristics were found.

### Correlation of vascular insulin receptor expression (VIR) with clinico-pathological patient characteristics

Next, we explored the putative biological significance of VIR. GCs with VIR-high were significantly more frequently localized at the proximal site and of unclassified phenotype according to Laurén. Interestingly, VIR status also correlated significantly with local tumor growth (T category), UICC stage, vessel invasion (V category), tumor budding, and the mucin phenotype (Table 1).

### HER2 status and the expression of the insulin receptor in gastric cancer

**Fig. 2:**
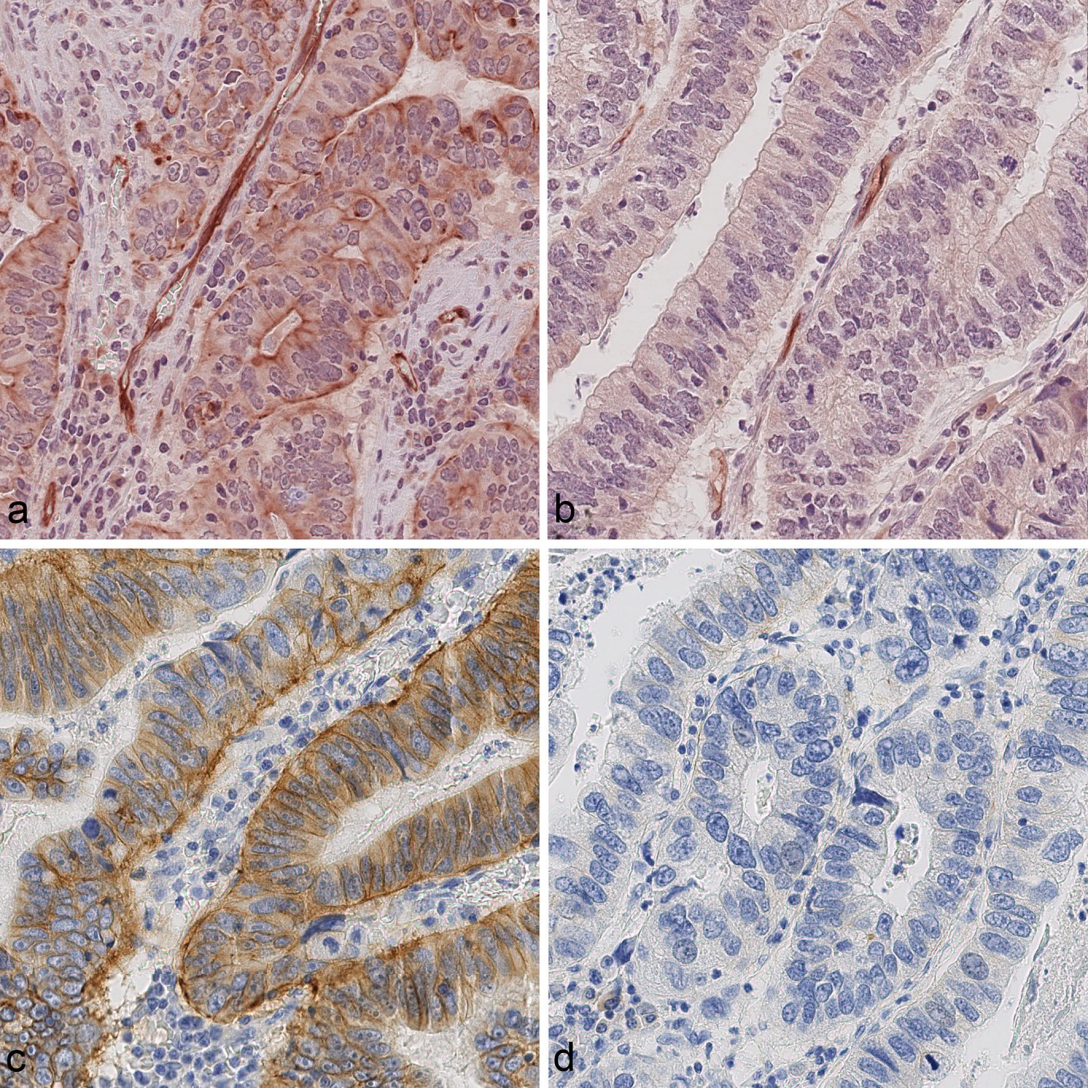
HER2 status and insulin receptor expression in gastric cancer. Gastric cancer sample showing areas of **a** high cytoplasmic (cCC-IR-high, HScore ≥ 90), high membranous (mCC-IR-high, HScore > 40), and high vascular (VIR-high, HScore ≥ 115) insulin receptor expression. The same sample contains areas, **b** of low cytoplasmic (cCC-IR-low, HScore < 90) and low membranous (mCC-IR-low, HScore ≤ 40) insulin receptor expression in tumor cells, while still showing high vascular (VIR-high, HScore ≥ 115) insulin receptor expression. The comparison with HER2 expression in the same tissue sample reveals co-localization with the areas of insulin receptor expression (**c**, **d**) no HER2 expression in the areas lacking insulin receptor expression in tumor cells. Anti-insulin receptor immunostaining (**a**, **b**), anti-Her2/neu-immunostaining (**c**, **d**); hematoxylin counterstaining. Original magnifications (**a**–**d**): 400×

Recently, it was shown that metformin may improve the prognosis that is associated with diabetes and insulin treatment, mainly in patients with primary HER2-positive and hormone receptor-positive breast cancer [15]. Therefore, we were interested to test the hypothesis that the IR status and the HER2 status are linked in GC. Interestingly, HER2-positive GCs were significantly more commonly cCC-IR-high (*p* = 0.002) and a trend was seen for mCC-IR-high (*p* = 0.073). No correlation was found between HER2 and VIR. Areas of cCC-IR-high and HER2 positivity were observed to overlap within the same tumor samples (Fig. 2).

### Survival analysis

**Fig. 3:**
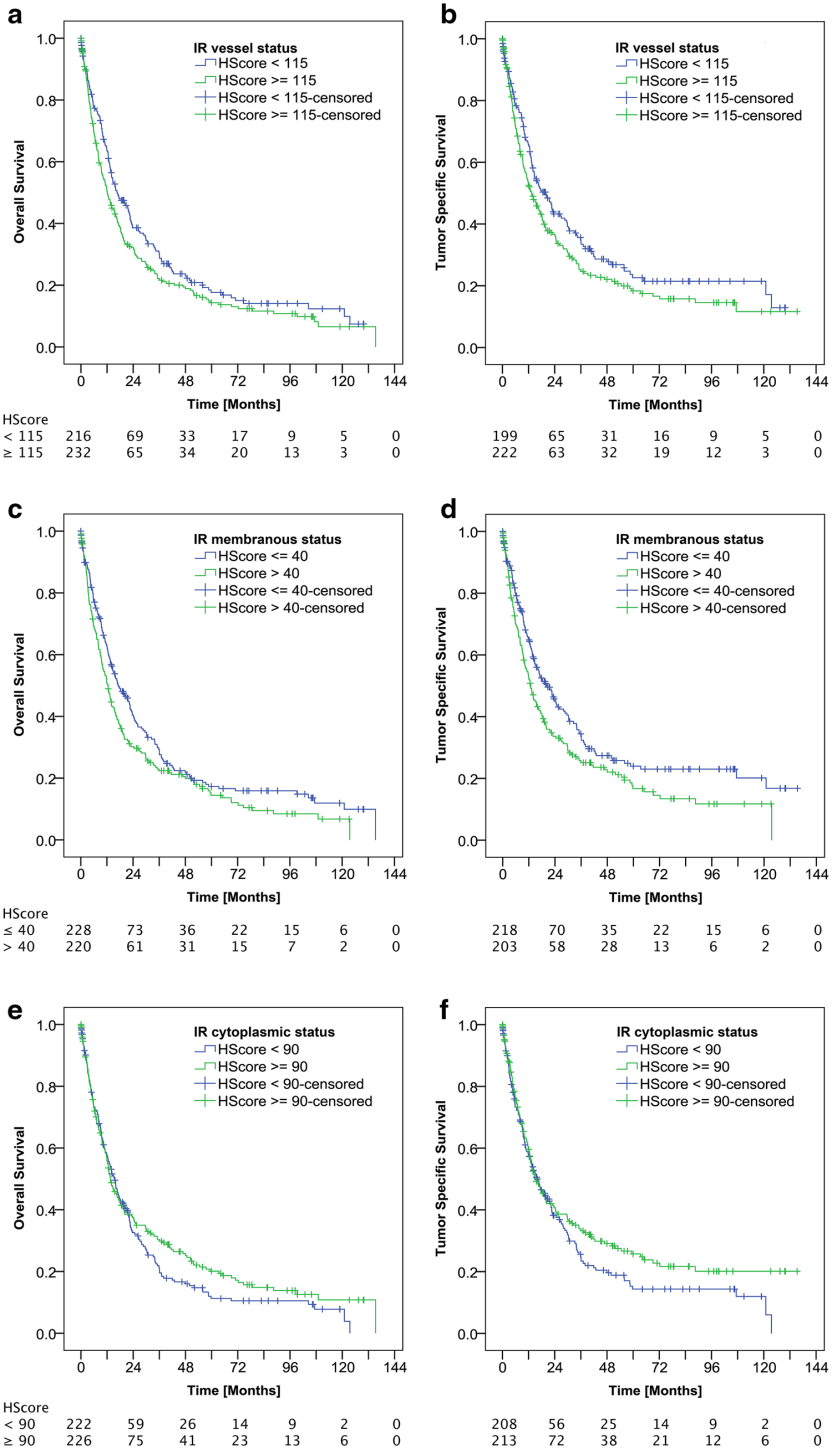
Kaplan–Meier curves demonstrating correlations between insulin receptor expression in tumor vasculature (VIR) and overall (**a***p* = 0.044; insignificant after multiple testing) as well as tumor-specific survival (**b***p* = 0.045; insignificant after multiple testing). Kaplan–Meier curves demonstrating correlations between membranous insulin receptor expression in tumor cells (mCC-IR) and overall (**c***p* = 0.026; insignificant after multiple testing) as well as tumor-specific survival (**d***p* = 0.01; significant after multiple testing). Kaplan-Meier curves demonstrating no correlations between cytoplasmic insulin receptor expression in tumor cells (cCC-IR) and overall (**e***p *= 0.214) as well as tumor specific survival (**f***p *= 0.135). Numbers at risk are provided below each Kaplan–Meier curve

The entire GC collective showed a median overall survival (OS) of 14.1 months and a median tumor-specific survival (TSS) of 15.5 months. Patient prognosis significantly depended on the Laurén phenotype, T, N, M, L, V, Pn, and R categories, UICC stage, lymph-node ratio, tumor budding (data not shown), VIR status, and mCC-IR status (Table 1; Fig. 3). Patients with VIR-high had a lower OS (12.1 months) and TSS (13.4 months) compared with the VIR-low group (OS: 16.7 months; TSS: 19.9 months), which lost significance after multiple testing (Table 1). Similarly, GCs with mCC-IR-high showed a median survival of 12.5 months (OS) and 12.8 months (TSS) compared with 17.3 (OS) and 20.3 months (TSS) of the mCC-IR-low group (Table 1). The correlation between mCC-IR and TSS remained significant after multiple testing (Table 1; Fig. 3). There was no significant correlation between cCC-IR and patient survival (Table 1). On multivariate analysis (Table 2), the VIR status turned out to be an independent prognosticator of overall (*p* = 0.010; hazard ratio = 1.355) and tumor-specific (*p* = 0.006; hazard ratio = 1.429) patient survival.

**Table 2:** Multivariate analysis

	Hazard ratio	95% CI	*p* value
*Overall survival*			
N category			0.005
N1 vs. N0	2.100	1.355–3.257	0.001
N2 vs. N0	1.707	1.022–2.851	0.041
N3 vs. N0	2.071	1.150–3.729	0.015
M category (M1 vs. M0)	1.671	1.242–2.249	0.001
Lymph-node ratio (high vs. low)	1.757	1.068–2.891	0.026
V category (V1 vs. V0)	1.526	1.086–2.143	0.015
Pn category (Pn1 vs. Pn0)	1.607	1.229–2.101	0.001
R status (R1/R2 vs. R0)	2.005	1.439–2.793	0.000
VIR status (high vs. low)	1.355	1.074–1.709	0.010
*Tumor-specific survival*			
N category			0.000
N1 vs. N0	2.430	1.451–4.069	0.001
N2 vs. N0	2.949	1.870–4.649	0.000
N3 vs. N0	4.381	2.865–6.701	0.000
M category (M1 vs. M0)	1.529	1.113–2.100	0.009
V category (V1 vs. V0)	1.642	1.147–2.350	0.007
Pn category (Pn1 vs. Pn0)	2.139	1.567–2.920	0.000
R status (R1/R2 vs. R0)	2.298	1.634–3.232	0.000
VIR status (high vs. low)	1.429	1.106–1.847	0.006

Independent variables after multivariate survival analysis (Cox regression, overall survival). All variables with *p* < 0.100 in univariate survival analysis (log-rank test) were included, i.e., the Laurén phenotype, local tumor growth (T category), nodal spread (N category), distant metastasis (M category), lymph-node ratio (LN ratio), lymphatic invasion (L category), vessel invasion (V category), grading (G category), resection status (R category), tumor budding (Bd category), perineural invasion (Pn category), membranous IR expression in tumor cells (mCC-IR), and vascular IR expression (VIR)

## Discussion

Our analysis of a large cohort shows for the first time that GCs express the IR in tumor cells (CC-IR) and endothelial cells of tumor vessels (VIR) and that both, CC-IR and VIR, are biologically relevant: CC-IR and VIR correlated with various clinico-pathological patient characteristics, including patient survival. VIR turned out to be an independent prognosticator of overall and tumor-specific patient survival on multivariate analysis. Interestingly, cCC-IR also correlated with the HER2 status, pointing towards a link between metabolic reprogramming of tumor cells and HER2.

IR was significantly more frequent in intestinal-type GC than in other GC types, such as determined by the Laurén classification and the mucin phenotype. This finding is in line with observations made previously by applying complex gene expression analyses. Using GC cell lines, Tan et al. [16] proposed two different intrinsic subtypes, an intestinal and a diffuse subtype. Genes up-regulated in the intestinal subtype were related to carbohydrate, protein metabolism, and cell adhesion, whereas cell proliferation and fatty acid metabolism were enriched in the diffuse subtype [16]. The significance of intrinsic subtypes was confirmed later-on by gene expression analyses: intestinal-type GCs more frequently showed the proliferative and metabolic gene signature [17]. The IR-expressing GCs of our collective matched several aspects of the proliferative subtype as described by Lei et al. [17]: the associations of IR expression with lower grading categories and higher patient age parallel the proliferative subtype’s features. Furthermore, in the proliferative subtype, RAS signaling and not epithelial–mesenchymal transition (EMT) [17] is supposed to be prevalent. This seems to be applicable to the IR-expressing tumor samples of our collective: first, the insulin receptor’s proliferative stimuli are known to be conveyed via RAS signaling [4], especially via its mitogenic isoform IR-A. Second, the morphologic manifestation of EMT [11] is considered to be tumor budding. IR expression was associated with low, but not with high tumor budding, thereby further supporting the notion that IR-expressing GCs rather belong to the proliferative than to the mesenchymal GC subtype. Nevertheless, IR expression was more frequent in samples with low than in samples without tumor budding, which might indicate that EMT might still play a limited role in IR-expressing GCs.

The biological effects of IR were observed to depend on its differential expression in tumor and endothelial cells, which was especially evident with respect to the T category. As VIR was associated with tumor size, we hypothesize that VIR might be involved in neoangiogenesis. This contention is supported by the observation that hypoxia up-regulates IR-expression in bladder cancer [18], and insulin induces endothelial cell tube formation and migration [19]. Against the background of the significantly diminished survival of VIR-high GC patients as demonstrated by our study, we, therefore, hypothesize that this patient group might benefit from IR-targeted therapies. In a translational medical approach, the drug metformin might prove to be useful in exploiting our findings of IR expression in GC.

Metformin is known to exhibit diverse indirect and direct antitumoral effects and influences a multitude of signaling pathways in cancer [20]. It suppresses IR and IGF1-receptor signaling indirectly by the reduction of the circulating ligands insulin and IGF1 and directly by interfering with the receptors’ signaling pathways [20]. Metformin has been shown to reduce the risk of developing GC [21, 22], to improve survival and to reduce cancer recurrence in GC patients with type 2 diabetes mellitus [23]. It would have been important to know the histological IR expression status of these patients, as diabetic hyperinsulinemic conditions are thought to promote cellular proliferation in cancer via the IR [24]. However, a correlation between diabetes mellitus and IR expression in GC has not been performed until now, and our findings of an association between VIR and worse survival in GC might be valid for non-diabetic patients, as well.

IR expression in GC tumor samples was observed to be heterogeneous, which fits to the concept of subclonal diversity in GC. We, therefore, accounted for tumor heterogeneity by applying a histology score, which enabled us to dichotomize between GC with predominantly elevated and GC with predominantly decreased IR expression. Tumor heterogeneity in GC has to be considered, as dominant cancer cell clones will eventually determine the clinical fate of the individual cancer patient—especially in the context of resistance to immunochemotherapy and the management thereof.

In terms of potential therapeutic implications, the significant correlation between cCC-IR and the HER2 status is highly intriguing: Upon IR stimulation, the activated IR is known to internalize from the cell surface, thereby resulting in cCC-IR [25]. Zhang et al. previously described a link between IR stimulation and resistance of HER2-positive GC against HER2-targeted therapy in vitro [26]. We, therefore, hypothesize that especially cCC-IR-positive GC patients with concomitant HER2 positivity might represent a new patient group, which could particularly benefit from metformin treatment. Highly encouraging in this respect are auxiliary findings in breast cancer: diabetic HER2-positive hormone receptor-positive breast cancer patients on metformin medication had an improved outcome under anti-HER2 therapy in a clinical trial [27] and in a retrospective study even without any specific anti-HER2 treatment [15]. The authors suspect an involvement of the IR/IGF1-receptor axis [15].

Summing up, we show that VIR and CC-IR are frequently expressed in GC. The expression is biologically significant and even correlates with the HER2 status, opening avenues for novel putative therapeutic interventions in GC.

## References

[1] Hanahan D, Weinberg RA (2011). Hallmarks of cancer: the next generation. Cell.

[2] Hur H, Paik MJ, Xuan Y, Nguyen DT, Ham IH, Yun J (2014). Quantitative measurement of organic acids in tissues from gastric cancer patients indicates increased glucose metabolism in gastric cancer. PLoS One.

[3] Kudou M, Kosuga T, Kubota T, Okamoto K, Komatsu S, Shoda K (2018). Value of preoperative PET-CT in the prediction of pathological stage of gastric cancer. Ann Surg Oncol.

[4] Vigneri R, Goldfine ID, Frittitta L (2016). Insulin, insulin receptors, and cancer. J Endocrinol Invest.

[5] Takahashi M, Inoue T, Huang M, Numakura K, Tsuruta H, Saito M (2017). Inverse relationship between insulin receptor expression and progression in renal cell carcinoma. Oncol Reports..

[6] Mulligan AM, O’Malley FP, Ennis M, Fantus IG, Goodwin PJ (2007). Insulin receptor is an independent predictor of a favorable outcome in early stage breast cancer. Breast Cancer Res Treat.

[7] Law JH, Habibi G, Hu K, Masoudi H, Wang MY, Stratford AL (2008). Phosphorylated insulin-like growth factor-i/insulin receptor is present in all breast cancer subtypes and is related to poor survival. Cancer Res.

[8] Kim JS, Kim ES, Liu D, Lee JJ, Solis L, Behrens C (2012). Prognostic impact of insulin receptor expression on survival of patients with nonsmall cell lung cancer. Cancer.

[9] Lauren P (1965). The two histological main types of gastric carcinoma: diffuse and so-called intestinal-type carcinoma. An attempt at a histoclinical classification. Acta Pathol Microbiol Scand..

[10] Brierley J, Gospodarowicz M, Wittekind C (2017). TNM classification of malignant tumours.

[11] Lugli A, Kirsch R, Ajioka Y, Bosman F, Cathomas G, Dawson H (2017). Recommendations for reporting tumor budding in colorectal cancer based on the International Tumor Budding Consensus Conference (ITBCC) 2016. Modern Pathol..

[12] Kemi N, Eskuri M, Ikalainen J, Karttunen TJ, Kauppila JH (2019). Tumor budding and prognosis in gastric adenocarcinoma. Am J Surg Pathol.

[13] Namikawa T, Hanazaki K (2010). Mucin phenotype of gastric cancer and clinicopathology of gastric-type differentiated adenocarcinoma. World J Gastroenterol.

[14] Warneke VS, Behrens HM, Boger C, Becker T, Lordick F, Ebert MP (2013). Her2/neu testing in gastric cancer: evaluating the risk of sampling errors. Ann Oncology..

[15] Kim HJ, Kwon H, Lee JW, Kim HJ, Lee SB, Park HS (2015). Metformin increases survival in hormone receptor-positive, HER2-positive breast cancer patients with diabetes. Breast Cancer Res.

[16] Tan IB, Ivanova T, Lim KH, Ong CW, Deng N, Lee J (2011). Intrinsic subtypes of gastric cancer, based on gene expression pattern, predict survival and respond differently to chemotherapy. Gastroenterology.

[17] Lei Z, Tan IB, Das K, Deng N, Zouridis H, Pattison S (2013). Identification of molecular subtypes of gastric cancer with different responses to PI3-kinase inhibitors and 5-fluorouracil. Gastroenterology.

[18] Roudnicky F, Dieterich LC, Poyet C, Buser L, Wild P, Tang D (2017). High expression of insulin receptor on tumour-associated blood vessels in invasive bladder cancer predicts poor overall and progression-free survival. J Pathol..

[19] Liu Y, Petreaca M, Martins-Green M (2009). Cell and molecular mechanisms of insulin-induced angiogenesis. J Cell Mol Med.

[20] Lei Y, Yi Y, Liu Y, Liu X, Keller ET, Qian CN (2017). Metformin targets multiple signaling pathways in cancer. Chin J Cancer..

[21] Zhou XL, Xue WH, Ding XF, Li LF, Dou MM, Zhang WJ (2017). Association between metformin and the risk of gastric cancer in patients with type 2 diabetes mellitus: a meta-analysis of cohort studies. Oncotarget..

[22] Tseng CH (2016). Metformin reduces gastric cancer risk in patients with type 2 diabetes mellitus. Aging (Albany NY)..

[23] Li P, Zhang C, Gao P, Chen X, Ma B, Yu D (2018). Metformin use and its effect on gastric cancer in patients with type 2 diabetes: a systematic review of observational studies. Oncol Lett..

[24] Belfiore A, Malaguarnera R (2011). Insulin receptor and cancer. Endocr Relat Cancer.

[25] Morcavallo A, Stefanello M, Iozzo RV, Belfiore A, Morrione A (2014). Ligand-mediated endocytosis and trafficking of the insulin-like growth factor receptor I and insulin receptor modulate receptor function. Frontiers Endocrinol..

[26] Zhang Z, Wang J, Ji D, Wang C, Liu R, Wu Z (2014). Functional genetic approach identifies MET, HER3, IGF1R, INSR pathways as determinants of lapatinib unresponsiveness in HER2-positive gastric cancer. Clin Cancer Res.

[27] Sonnenblick A, Agbor-Tarh D, Bradbury I, Di Cosimo S, Azim HA, Fumagalli D (2017). Impact of diabetes, insulin, and metformin use on the outcome of patients with human epidermal growth factor receptor 2-positive primary breast cancer: analysis from the ALTTO phase III randomized trial. J Clin Oncol.

